# *Halobacteriovorax* halts disease progression in endangered Caribbean corals

**DOI:** 10.1093/ismejo/wraf270

**Published:** 2025-12-10

**Authors:** Lauren Speare, Chloe Manley, Sunni Patton, Eddie Fuques, Macey N Coppinger, Rebecca L Vega Thurber

**Affiliations:** School of Biological Sciences and Center for Microbial Dynamics and Infection, Georgia Institute of Technology, 950 Atlantic Drive NW, Atlanta, GA 30332, United States; School of Biological Sciences and Center for Microbial Dynamics and Infection, Georgia Institute of Technology, 950 Atlantic Drive NW, Atlanta, GA 30332, United States; Marine Science Institute, University of California at Santa Barbara, Building 520, Santa Barbara, CA 93106, United States; Marine Science Institute, University of California at Santa Barbara, Building 520, Santa Barbara, CA 93106, United States; School of Biological Sciences and Center for Microbial Dynamics and Infection, Georgia Institute of Technology, 950 Atlantic Drive NW, Atlanta, GA 30332, United States; Marine Science Institute, University of California at Santa Barbara, Building 520, Santa Barbara, CA 93106, United States

**Keywords:** probiotic, scleractinian, Vibrio, disease-model, Acropora

## Abstract

Predation is a top-down regulator of ecosystem integrity and a key driver of community structure and evolution in plants and animals. Despite our awareness of these dynamics, our understanding of microbial top-down control by bacterial predators remains limited. Predatory *Halobacteriovorax* bacteria are common, low abundance members of many marine and estuarine microbiomes and are considered generalists with less specific prey ranges than most viruses, yet more selective targets than antibiotics. This “Goldilocks” prey range has huge potential to treat polymicrobial infections, particularly in complex microbiomes; however, few studies employing *Halobacteriovorax* as a tool to manipulate dysbiotic microbiomes have been pursued. We developed a single-pathogen disease mitigation model in the critically endangered Caribbean coral, *Acropora cervicornis.* We employed a strain of the highly versatile *Vibrio coralliilyticus* as our pathogen, which causes rapid tissue loss and death in stony corals and mortality in oyster larvae. To demonstrate that predatory bacteria can alter disease dynamics in corals we infected *A. cervicornis* with virulent *V. coralliilyticus* and upon the first signs of disease, treated corals with *Halobacteriovorax* cultures. Without predators, 100% of corals were bleached by 48 h and 86% displayed tissue loss within five days; however with *Halobacteriovorax,* 57% of corals did not bleach beyond the inoculation site and no tissue loss was observed. This living probiotic successfully halted *Vibrio-*induced disease progression in *A. cervicornis*, suggesting that predatory bacteria broadly function as top-down regulators of community dynamics in eukaryotic microbiomes and microbial predators are a promising coral disease therapy.

## Introduction

Scleractinian corals are facing an unprecedented global decline due to the impacts of anthropogenic climate change which are only predicted to increase. Disease is a primary contributor to coral mortality and both the prevalence and severity of disease have increased dramatically over the past several decades [1–3]. The Caribbean, commonly referred to as a disease “hot spot,” has the highest reports of disease globally [4, 5] and has been ravaged by numerous diseases including “white” diseases (white band I and II, white plague I, II, and III, white pox) [6–11] and stony coral tissue loss disease [12]. Broad-spectrum antibiotics can avert disease onset and progression for individual corals and their use, in combination with mechanical diseased tissue removal, is becoming more common on Caribbean reefs. For example, studies on diseased corals in Florida and across the Caribbean [13–16] revealed that a combination of amoxicillin and Base 2B silicone paste can quickly halt SCTLD progression, making it an attractive disease intervention. However, antibiotic usage for corals has raised concerns regarding rapid antibiotic resistance development, host gene expression modulation, and unintended microbiome changes [17–20]. These concerns highlight the need for additional disease treatments.

Ongoing research efforts aim to develop long-term solutions to identify and selectively breed more disease tolerant/resistant genotypes as well as short-term treatments to preserve current biodiversity [21–25]. Despite these efforts, effective strategies to prevent and treat disease are still limited. This limitation stems, in part, because the majority of described coral diseases lack a well-defined etiological agent, many diseases are thought to be polymicrobial, and only a handful of discrete coral pathogens have been confirmed [26–29]. *Vibrio coralliilyticus* is one of the few well-described pathogens that infects a wide range of coral hosts [30–36]. *V. coralliilyticus* is a primary pathogen [30, 31, 36] and can also contribute to polymicrobial infections [37–43]. Thus, short-term disease prevention and treatment strategies should ideally include both broad-spectrum approaches, to target many potential disease agents, as well as selective approaches, to discriminate between microbes to eliminate (pathogens and opportunists) and those to avoid (beneficial symbionts).

Biological treatments such as inoculations of single taxa or consortia of beneficial microorganisms for corals (BMCs) [44] have the potential nuance that broad-spectrum antibiotic and mechanical treatments lack. Such taxa may protect coral through niche occupation, diffusible small molecule production [45–47], direct antagonism [48–50], and other mechanisms [51]. BMCs containing many unique taxa may promote overall coral resilience through multiple mechanisms [44] including disease mitigation [48], whereas single-taxa inoculation studies are far less explored, yet show promise to prevent and treat disease [29]. For example, a recent study showed that *Ruegeria profundi* prevented *Vibrio*-induced bleaching, tissue loss, and microbiome alterations when administered after *Vibrio* inoculation yet prior to visible bleaching [52]. Another study revealed that *Pseudoalteromonas* McH1–7 can protect *Montastraea cavernosa* from SCTLD when administered prophylactically, and can slow the spread of SCTLD between untreated corals in close proximity with treated corals [45].

One area of study that has gotten less attention is the use of natural predators of disease-causing bacteria. Marine predatory bacteria are potentially powerful single-taxa coral probiotics due to their unique mechanism of action: direct consumption of prey as their primary food source. *Bdellovibrio* and like organisms (BALOs) phylum Bdellovibrionota, are generalist predators that prefer nutrient-responsive bacterial species, including many pathogens such as *Vibrio, Pseudomonas, Salmonella,* and *Escherichia* [53]. Marine BALOs also consume copiotrophs and several studies have shown marine BALOs can effectively reduce *Vibrio* abundance in aquaculture [54–57]. *Halobacteriovorax* are a marine genus of obligate bacterial predators that are natural, low abundance members of marine biofilms [58, 59] and coral microbiomes [60]. In a previous study, *Halobacteriovorax* prevented *Vibrio*-induced infections without altering the rest of the microbiome when predator and prey were administered simultaneously [49]. Thus, *Halobacteriovorax* has the potential to act as a top-down structuring force to modulate coral microbial communities, although whether this mechanism can treat established diseases is unknown. Therefore, this new work aimed to investigate the application of marine predatory bacteria as a disease treatment strategy. In doing so, we also evaluated the native predator community and how it is shaped by a foreign predator.

## Materials and Methods

### Coral collection, husbandry, and health assessment

Coral husbandry and experiments were performed at the Mote’s Elizabeth Moore International Center for Coral Reef Research and Restoration (IC2R3) in Summerland Key, Florida from February to March 2023. Experiments were performed in 2.5 L aquaria maintained at 27.5°C with locally sourced sea water filtered for sand and large particles from the Atlantic side of the keys. Aquaria were housed outside under natural light regimes with ~75% shade to account for shallow aquarium depth and kept in a flow-through seawater table allowing for consistent temperature regulation between aquaria. Each tank contained a circulation pump to keep water moving and water was changed daily (~25% water volume). Header tank pH was stabilized at ~8.0 by aeration and a venturi pump system.

Two reef-building Caribbean coral species with distinct morphologies and disease histories were selected for this work. The branching coral *A. cervicornis* is a critically threatened species that is highly susceptible to a variety of Acropora White Syndromes [29, 61]. We selected a disease susceptible genotype (ML-50) [62] for this work to enhance the likelihood of developing a successful disease model. We also previously used this coral genotype to study a variety of host–microbe interactions [17, 63–65]. To expand our model’s utility we also used *Pseudodiploria strigosa,* a widely distributed species of brain coral that is highly susceptible to stony coral tissue loss disease [66–69]; this genet had unknown disease susceptibility/resistance. Corals ~5 cm in diameter were collected from the Mote’s *in situ* coral nursery in Looe Key in February 2023, attached to concrete plugs, and acclimated to aquarium conditions for 3 weeks. For each experiment, fragments were randomly assigned treatment groups and locations within aquaria. Coral fragments were visually assessed using the Coral Health Chart, a standardized color reference card [70–73], by three independent observers at each time point.

### Planktonic/broth infection assay (Fig. 1, 2A, and S1)

**Figure 1:**
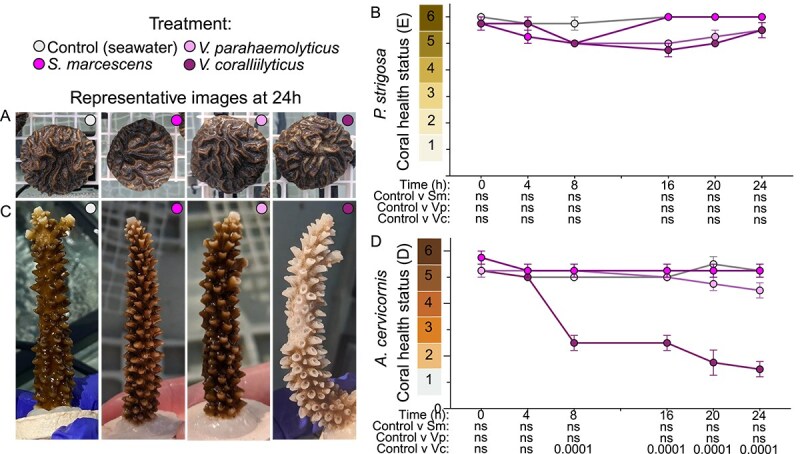
*V. coralliilyticus* RE22 induces bleaching in *A. cervicornis* ML-50 yet not *P. strigosa*. Corals were exposed to sterile seawater (control; gray), or one of three bacterial cultures: *S. marcescens* ATCC 13880 (medium pink), *V. parahaemolyticus* MC_102 (light pink), or *V. coralliilyticus* RE22 (dark pink). Data are shown as representative images taken 24 h after bacterial inoculation (A: *P. strigosa*; C: *A. cervicornis*) or average coral health chart score across all fragments in each treatment (B: *P. strigosa*; D: *A. cervicornis*). Results of two-way ANOVA with Dunnett’s multiple comparison test for each bacterial inoculum against the control are shown for each time point below the x-axis. Four coral fragments were used in each treatment (*n =* 32); error bars indicate SEM; ns indicates “not significant.”

**Figure 2:**
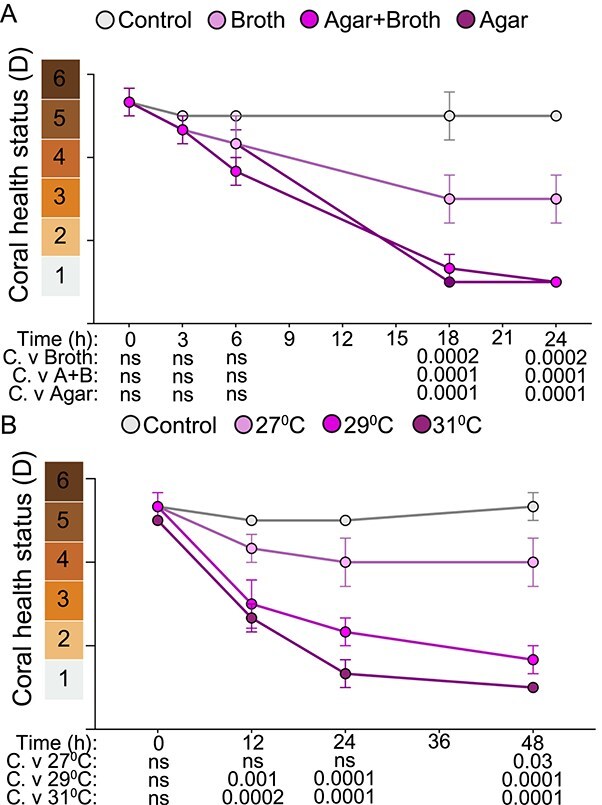
Rate of rapid *V. coralliilyticus* bleaching is dependent on temperature and inoculation method. Pigmentation of *A. cervicornis* exposed to *V. coralliilyticus* inoculated via different methods (A) or grown at varying temperatures (B). Data are shown as the average coral health chart score across all fragments in each treatment. Results of two-way ANOVA with Dunnett’s multiple comparison test for each treatment against the control (C.) are shown for each time point below the x-axis. Three coral fragments were used in each treatment (*n =* 24); error bars indicate SEM; ns indicates “not significant.”

To induce disease phenotypes, coral fragments were exposed to three bacterial cultures: *Serratia marcescens* MC_101, *V. parahaemolyticus* MC_102, and *V. coralliilyticus* and MC_103*.* Whole genomes of each strain were sequenced on an Illumina NovaSeq X Plus sequencer and three housekeeping genes (*recA, mdh,* and *pyrC*) were blasted against the NCBI database to determine strain identity. *S. marcescens* MC_101 and *V. coralliilyticus* MC_103 had a perfect match to *S. marcescens* ATCC 13880 (CP071206) and *V. coralliilyticus* RE22 (PRJNA482426) [74], respectively, and are thus considered the same strains. *V. parahaemolyticus* MC_102 did not perfectly match any strain in the database; *recA* and *pyrC* sequences matched *V. parahaemolyticus* strain Colony 547 (PRJNA668870) at 100%, but *mdh* more closely matched to strain 2013V-1136 (PRJNA266293). *V. coralliilyticus* RE22 can induce tissue loss in *Astrangia poculata* [75] yet does not readily induce disease in *Montipora capitata* [76]. *V. coralliilyticus* as a species infects a broad range of corals, including other *Acropora* spp.*,* and individual strains may show host specificity [32, 34, 35, 75–77]. The other strains have not been previously linked to coral disease; however other strains of these species have. *S. marcescens* is the causative agent of acroporid serratiosis (White Pox) in *Acropora palmata* [78]; *V. parahaemolyticus* has been implicated in Porites ulcerative White Spot Disease [79].

Heterotrophic bacterial strains were revived fresh from −80°C stocks by streaking onto LBS (Luria Bertani with added salt [20 g NaCl/L]) agar plates, restreaking once, and starting liquid cultures from single colonies. Overnight liquid cultures were subcultured and grown in LBS at 31°C, shaking at 115 rpm until early mid exponential phase (OD600 of 0.16–0.4). Cells were washed three times with filter sterilized instant ocean (FSIO [25 ppt IO]) to remove excess nutrients and resuspended into fresh FSIO. The resulting cell suspension was quantified by plating serial dilutions onto LBS agar plates [80]. Each fragment was transferred to a 500 ml beaker containing ~10^6^ colony forming units (CFUs) per ml of a single bacterial strain (Table 1, Fig. 1). Initial tests showed that bacterial inoculation at ambient aquarium temperatures (27.5°C) was insufficient to induce disease phenotypes, therefore corals were exposed to each heterotrophic strain at 31°C for 1 h and returned to 2.5 L aquaria at 27.5°C for monitoring (Table 1). Control corals were transferred into beakers containing sterile FSIO and exposed to 31°C for 1 h. Coral fragments were visually assessed using the Coral Health Chart, a standardized color reference card [70–73], by three independent observers at each time point. A second trial with *P. strigosa* was performed using a higher dose inocula (~10^7^–10^10^ CFUs/ml, Fig. S1).

**Table 1:** Microbial inoculum for each experiment; CFUs from control treatments were either at or below the limit of detection (2×10^2^ CFUs/ml). Experiments described in Fig. 1, S1, and 3 had four technical replicates to quantify bacterial abundance per treatment, and experiments described in Fig. 2 had three technical replicates per treatment; data are shown as Average (+/− standard deviation). “Broth” indicates planktonic addition.

Figure	Treatment	Inoculation method	Concentration of microbial cells (CFUs/ml)
Figure 1	*V. coralliilyticus*	Broth	1.5 (± 1.5) ×10^6^
	*V. parahaemolyticus*	Broth	4.0 (± 1.1) ×10^6^
	*S. marcescens*	Broth	2.0 (± 1.3) ×10^6^
Figure S1	*V. coralliilyticus*	Broth	9.0 (± 3.5) ×10^7^
	*V. parahaemolyticus*	Broth	5.0 (± 1.7) ×10^10^
	*S. marcescens*	Broth	8.7 (± 1.3) ×10^9^
Figure 2A	*V. coralliilyticus*	Agar pad	1.3 (± 0.4) ×10^6^
	*V. coralliilyticus*	Broth	7.6 (± 0.8) ×10^5^
Figure 2B	*V. coralliilyticus* (27°C)	Agar pad	4.8 (± 1.2) ×10^7^
	*V. coralliilyticus* (29°C)	Agar pad	6.0 (± 2.4) ×10^7^
	*V. coralliilyticus* (31°C)	Agar pad	5.2 (± 1.8) ×10^7^
Figures 3–5	*V. coralliilyticus*	Agar pad	7.5 (± 1.1) ×10^7^
	*Halobacteriovorax sp.*	Broth	1.3 (± 0.5) ×10^5^

### Agar pad infection assay (Fig. 2 and 3)

**Figure 3:**
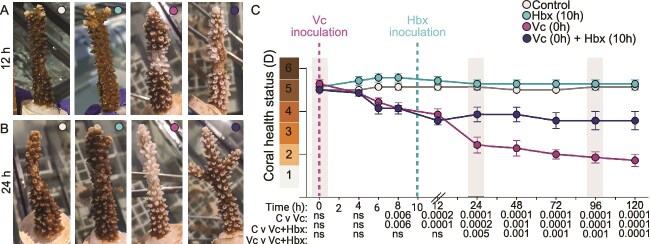
*Halobacteriovorax* treatment halts disease progression in *A. cervicornis*. Corals were exposed to sterile seawater (control [C]; gray), or one of three bacterial treatments at various time points: *Halobacteriovorax sp. GFR8* (Hbx) at 10 h (teal); *V. coralliilyticus* RE22 (Vc) at 0 h (pink); or *V. coralliilyticus* RE22 at 0 h and then *Halobacteriovorax sp*. *GFR8* at 10 h (blue). Data are shown as representative images taken at 12 h (A) or 24 h (B) after the start of the experiment or as the average coral health chart score across all fragments in each treatment (C). Results of two-way ANOVA with Dunnett’s multiple comparison test for each bacterial treatment against the control or *V. coralliilyticus* only versus *V. coralliilyticus + Halobacteriovorax* (Vc v Vc + Hbx) are shown for each time point below the x-axis. Seven coral fragments were used in each treatment (*n =* 28); error bars indicate SEM; ns indicates “not significant.” Gray bars indicate polyp sampling for microbiome analysis (later figures).

*V. coralliilyticus* RE22 cultures were revived, grown to mid exponential phase, and washed as described above. To embed cultures into agar pads, cells were pelleted by centrifugation at 10000 rpm for 3 min, supernatant was poured off, and cells were resuspended in 600 μl of molten low melt agar. Low melt agar was warm to the touch but comfortable to hold to ensure that it did not scald cells. While the *Vibrio*-embedded low melt agar cooled, coral fragments were removed from aquaria and patted dry with kimwipes. Once the *Vibrio-*embedded low melt agar began to solidify, 600 μl of low melt agar was carefully pipetted onto each coral, approximately halfway up the fragment. Corals remained exposed to the air for 30 s to allow agar pad to fully solidify and were then returned to FSIO in beakers. After 1 h, agar pads were removed by gently jostling each fragment. Control corals received a sterile low melt agar pad. The concentration of *V. coralliilyticus* within representative agar pads was quantified by plating serial dilutions onto LBS agar plates [80].

Temperature range experiments with *V. coralliilyticus* RE22 (Fig. 2B) were performed with the following modifications. Overnight liquid cultures of *V. coralliilyticus* RE22 were grown in LBS at either 27°C, 29°C, or 31°C, shaking at 115 rpm until early mid exponential phase (OD600 of 0.16–0.4). Cultures were washed, resuspended in FSIO, quantified, and embedded into low melt agar as described above. Corals were inoculated with agar pads of each culture and then placed into beakers at the corresponding temperature of *V. coralliilyticus* RE22 growth at for 1 h. Control corals were inoculated with sterile agar pads and held at 31°C for 1 h. After agar pad removal, each coral was returned to 2.5 L aquaria at 27.5°C.

### *Halobacteriovorax sp.* GFR8 inoculation assay (Fig. 3)

*Halobacteriovorax sp.* GFR8 was isolated as described previously [81] and grown on liquid cultures of *V. parahaemolyticus* in PP25 medium (1 g/L polypeptone and 25 ppt IO) at 28°C, shaking at 85 rpm. Cultures were revived from −80°C stocks and allowed to grow for 2 days until prey clearing was observed. Cultures were then subcultured into fresh prey and upon clearing passed through a 0.45 μm filter to remove the larger prey cells; the resulting filtrate was used for coral inoculations. *Halobacteriovorax sp*. cultures were quantified by performing serial dilutions of filtrate and plating dilutions onto lawns of *V. parahaemolyticus* prey. Approximately 10 h after *V. coralliilyticus* RE22 agar pad inoculation, corals were removed from 2.5 L aquaria and transferred to beakers containing either filter-sterilized IO or filtered *Halobacteriovorax sp.* GFR8 cultures (Table 1). Corals were incubated at 27.5°C for 3 h oscillating at 85 rpm and then transferred back into aquaria. After day one of the experiment, coral fragments were visually assessed for pigmentation at 24-h intervals.

### DNA extraction and sequencing

Coral tissue was collected from randomly selected polyps that reflected the pigmentation of the entire fragment at the midpoint of the coral fragment. Coral polyps were collected at three time points: prior to the start of the experiment, at 24 h, and at 96 h, totaling nine polyps from each coral fragment, resulting in 84 coral tissue samples. Coral polyps were collected using ethanol-sterilized bone cutters and tissue was immediately placed into DNA/RNA shield and stored at −80°C until further processing. Each sample (coral polyp and DNA/RNA shield), and an additional six negative control samples (three MilliQ samples and three DNA/RNA shield samples) were extracted for DNA using the DNeasy PowerSoil Pro Kit according to manufacturer instructions (Qiagen). Extracted DNA was purified by DNA Clean and Concentrator Kits (Zymo). Samples were submitted to the Parker H. Petit Institute for Bioengineering and Bioscience High Throughput sequencing core at the Georgia Institute of Technology for library preparation, 2-step PCR amplification, and sequencing on a MiSeq System (Illumina). The 16S gene V4 region was targeted using 515FB (5′TCGTCGGCAGCGTCAGATGTGTATAAGAGACAGGTGYCAGCMGCCGCGGTAA-3′) [82] and 806RB (5′GTCTCGTGGGCTCGGAGATGTGTATAAGAGACAGGGACTACNVGGGTWTCTAAT-3′) [83] primers. One sample (from Vc-only at 24 h) was not able to be sequenced and was not included in downstream analysis.

### Microbiome analysis

16S rRNA amplicon data were analyzed in R unless otherwise stated. An initial quality check was performed on demultiplexed samples, all of which passed the minimum quality threshold. The DADA2 package was used to remove (truncate) low quality nucleotides from each read (last five nucleotides), merge paired reads, construct a sequence table, and remove chimeras [84]. Phyloseq was used to assign taxonomy, decontaminate samples, and evaluate beta diversity [85]. Taxonomy was assigned using the Silva 132 database (as of July 2024) and identified 9823 ASVs. Samples were decontaminated for ASVs that were more prevalent (3-fold or greater) in the negative control samples (MilliQ and DNA/RNA Shield) compared to the coral samples (57 ASVs), and eukaryotic ASVs (235 ASVs). Samples were then rarefied using the vegan package [86] at 45,000 reads per sample for downstream diversity analyses (richness and relative abundance comparisons). Rarefying removed 843 additional ASVs, resulting in 8688 total ASVs remaining. Differential abundance analysis on the entire community, including abundant and rare (predator) taxa, was performed on unrarefied data using DESeq2 [87]. Data visualization was performed either in R, Graphpad Prism, or biorender.

## Results

### *V. coralliilyticus* RE22 infection promotes bleaching in *A. cervicornis* ML-50

To begin exploring the potential of *Halobacteriovorax* as a coral probiotic, we first needed to identify a heterotrophic bacterium that could induce disease phenotypes in our Caribbean corals. Corals were exposed to one of three heterotrophic cultures, or sterile control conditions. Control corals maintained a dark pigmentation throughout the experiment (Fig. 1). We did not observe paling or bleaching in the *P. strigosa* fragments included in this (Fig. 1A and 1B) or a second trial of this experiment (Fig. S1; Table 1). For *A. cervicornis,* there was no significant decrease in pigmentation in fragments exposed to *S. marcences* ATCC 13880 (MC_101) or *V. parahaemolyticus* MC_102. However, in *A. cervicornis* exposed to *V. coralliilyticus* RE22 (MC_103) there was significant paling after 24 h that then progressed to bleaching and tissue loss after several days (Fig. 1C and D). We also observed a decrease in seawater clarity in *V. coralliilyticus-*treated *A. cervicornis* tanks, likely due to dinoflagellate and/or mucus expulsion from coral tissue into the surrounding water. These observations suggest *A. cervicornis* ML-50 is susceptible to infection and disease by *V. coralliilyticus* RE22*,* and these signs are dependent specifically on this bacterium*,* rather than simply introduction of any foreign bacterium at high concentrations.

### *V. coralliilyticus* RE22-induced bleaching is temperature dependent

We optimized our infection method by concentrating the site of inoculation to a single location along the coral fragment. We did not observe any loss of pigmentation in corals exposed to bacteria-free low-melt agar (Fig. 2A). At 6 h we observed paling where the *V. coralliilyticus*-containing agar pad was placed on the coral fragment, yet not throughout the entire fragment. This paling progressed to bleaching that radiated upward along the fragment within 24 h (Fig. 2A). Corals exposed to this pathogen-embedded agar pad achieved the same level of bleaching as corals exposed to similar concentrations of the planktonic pathogen in seawater (Table 1, Fig. 2A), suggesting both methods are sufficient to induce bleaching under the conditions tested.

Because virulence of some *V. coralliilyticus* strains is highly temperature-dependent [30], and in hopes of minimizing unnecessary temperature stress, we tested whether elevated temperatures were necessary to elicit active infection in *A. cervicornis* by *V. coralliilyticus* RE22*.* We grew RE22 cultures at 27°C, 29°C, or 31°C and exposed corals to ~10^7^ cells per ml in an agar pad (Table 1) for 1 h at 27°C, 29°C, or 31°C, respectively. As a control, we exposed a subset of coral fragments to 31°C for 1 h with a bacteria-free agar pad to ensure any observed paling or bleaching was dependent on activity by RE22 and not an acute increase in temperature or the agar itself. We observed a significant decrease in pigmentation and bleaching at the polyp tips for corals in each pathogen treatment compared to the control by 24 h (Fig. 2B). Corals in the 27°C treatment were slower to palethan the other temperature treatments and did not progress to bleaching (Fig. 2B). Corals in the 29°C and 31°C treatments showed significant paling at 12 h and continued to lose pigmentation throughout the experiment, fully bleaching by 48 h (Fig. 2B). These data suggest that *V. coralliilyticus* RE22 can induce bleaching in *A. cervicornis* in a temperature-dependent manner, similar to other pathogenic *V. coralliilyticus* strains [88], and temperature during pathogen growth and exposure can significantly alter the rate and extent of bleaching. With these observations in mind, we chose to conduct the following coral disease experiment using this pathogen-embedded agar method at 31°C.

### *Halobacteriovorax sp.* GFR8 inoculation halts bleaching progression by *V. coralliilyticus* RE22

Employing this *A. cervicornis* single-pathogen disease system, we sought to determine whether the predatory bacterium *Halobacteriovorax sp.* GFR8 was able to mitigate the effects *V. coralliilyticus* RE22-induced tissue loss. Based on previous results [49], we predicted that GFR8 inoculation after initial disease onset could diminish both RE22-induced coral microbiome shifts and disease progression. To test this prediction, we split corals into four different inoculation groups: no inoculation control (Control), *V. coralliilyticus* RE22 only (Vc-only), *V. coralliilyticus* RE22 followed by *Halobacteriovorax sp.* GRF8 (Vc + Hbx), and *Halobacteriovorax sp.* GRF8 only (Hbx-only) (Fig. 3). Vc-only and Vc + HBX corals were exposed to *V. coralliilyticus*-embedded agar pads at the beginning of the experiment (Time = 0 h) for 1 h (Table 1). Shortly after the first signs of paling, ~10 h after the start of the experiment (Time = 10 h) we exposed Vc + Hbx corals and Hbx-only corals to *Halobacteriovorax sp.* GRF8 cultures for 3 h and then returned them to aquaria to monitor for the remainder of the experiment.

Control and Hbx-only corals did not display any bleaching and maintained dark pigmentation throughout the experiment (Fig. 3). The majority of Vc-only and Vc + Hbx corals showed the first signs of bleaching ~8 h after the start of the experiment and showed significant pigmentation loss at the location of the agar pad (11 out of 14 fragments) (Fig. 3C). At this time, we noticed a decrease in seawater clarity in these tanks and a visible increase in expelled coral mucus. By 12 h, the majority of Vc-only and Vc + Hbx corals had paled throughout the entire fragment and bleached at the tips of each polyp (13 out of 14 fragments) (Fig. 3A). For Vc-only corals, this paling progressed into bleaching by ~48 h (6 out of 7 fragments) and tissue loss by ~120 h (6 out of 7 fragments) (Fig. 3B and C). However, for Vc + Hbx corals, we did not observe any significant decrease in pigmentation after 12 h (Fig. 3C). Vc + Hbx corals showed significantly less bleaching compared to Vc-only corals and did not experience any tissue loss yet remained significantly paler than Control or Hbx-only corals (Fig. 3), which is not surprising given the short duration of this experiment (5 days). Taken together, these data suggest that inoculation of GFR8 upon the first signs of RE22-induced bleaching can halt disease progression in *A. cervicornis* genotype ML-50.

### Bacterial inoculation shifts *A. cervicornis* microbiome structure

**Figure 4:**
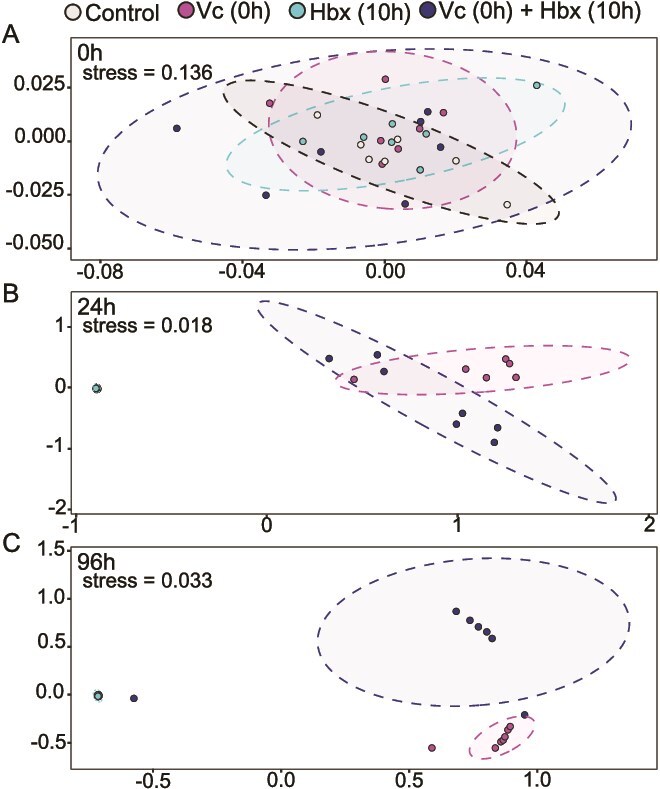
*V. coralliilyticus* and *Halobacteriovorax* shift *A. cervicornis* microbiome structure. Non-metric multidimensional scaling (NMDS), based on Bray–Curtis dissimilarity between samples from the *V. coralliilyticus-Halobacteriovorax* inoculation experiment (Fig. 3). Symbol color indicates sample treatment: control corals inoculated with only seawater (gray); corals inoculated with *V. coralliilyticus* RE22 at 0 h (pink); corals inoculated with *Halobacteriovorax sp*. GFR8 at 10 h (teal); corals inoculated with *V. coralliilyticus* RE22 at 0 h and *Halobacteriovorax sp*. GFR8 at 10 h (blue). Data are shown at the start of the experiment prior to inoculation (A); 24 h (B), and 96 h later (C). NMDS1 is shown on the x-axis and NMDS2 is shown on the y-axis. Each data point represents the microbial community composition of one sample; ellipses represent 95% confidence intervals; stress values are shown on each panel.

Given the significant effect of *V. coralliilyticus* RE22 and *Halobacteriovorax sp*. GFR8 inoculation on bleaching progression*,* we predicted that coral microbiomes in these treatments would also be impacted. Non-metric multidimensional scaling (NMDS) plots of 16S rRNA gene amplicons revealed a significant effect of RE22 on bacterial communities (Fig. 4). At the beginning of the experiment, all treatment groups overlapped substantially, indicating no differentiation in community composition (Fig. 4A). By 24 h, Control and Hbx-only samples clustered tightly together, yet separately from Vc-only and Vc + Hbx samples which overlapped with each other (Fig. 4B; permanova; *R*^2^ = 0.80, *P* < .001). Pairwise permanova indicated that Vc-only and Vc + Hbx communities differed significantly from Control and Hbx-only (*P* < .012). By 96 h, Vc-only and Vc-Hbx samples clustered distinctly from one another (Fig. 4C; permanova, *R*^2^ = 0.77, *P* < .001; pairwise permanova, *P <* .012), suggesting further differentiation in their community composition over time. Beta dispersion did not significantly differ for the Control or Hbx-only samples throughout the experiment. For the Vc-only samples, beta dispersion significantly increased from 0 h to 24 h (permanova; *P* < .001) but did not significantly change from 24 to 96 h. For Vc + Hbx samples, beta dispersion significantly increased from 0 h to 24 h and 24 h to 96 h (permanova; *P* < .001), reflecting a sustained increase in community variation over time.

### *V. coralliilyticus* RE22 inoculation significantly increases alpha diversity by reducing *Rickettsiales* relative abundance

**Figure 5:**
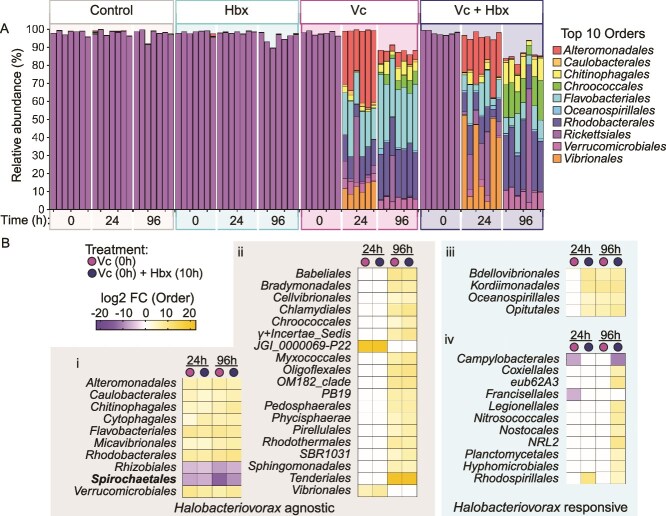
*V. coralliilyticus* and *Halobacteriovorax* shift *A. cervicornis* microbiome structure. Relative abundance (%) of microbiome samples from the *Vibrio-Halobacteriovorax* inoculation experiment (Fig. 3). Data are shown as the 10 most abundant orders (A). Background color indicates the sample treatment: control corals inoculated with only seawater (gray); corals inoculated with *Halobacteriovorax* at 10 h (teal); corals inoculated with *V. coralliilyticus* at 0 h (pink); corals inoculated with *V. coralliilyticus* at 0 h and *Halobacteriovorax* at 10 h (blue). Data are shown for individual samples, with 6–7 samples in each treatment (*n =* 83). (B) Heatmap displaying results in the form of log2 fold change (log2 FC) from order-level differential abundance analysis. White boxes indicate nonsignificant values. Orders are organized by their response to *Halobacteriovorax*: “*Halobacteriovorax* agnostic” (gray box on the left) indicates orders showed the same differential abundance trend in both *V. coralliilyticus* and *V. coralliilyticus + Halobacteriovorax* treatments; “*Halobacteriovorax* responsive” (teal box on the right) indicates orders that showed a unique differential abundance pattern between Vc-only and Vc-Hbx treatments. Statistical analyses were performed via DESeq2 and results are shown in Table S2.

To determine how bacterial inoculations altered coral microbiomes, we generated stacked bar graphs of the top 10 most abundant bacterial orders within our samples (Fig. 5A). There were no significant relative abundance differences at the order level at the beginning of the experiment between any treatments. Unsurprisingly, *Rickettsiales* was the dominant member of the microbiome (97.9% on average; Table S1) in all samples, consistent with other observations for this genotype [63, 64]. *Rickettsiales* did not significantly change in relative abundance for Control or Hbx-only corals throughout the experiment (Fig. 5A, Table S1). *Rickettsiales* relative abundance did significantly decrease in Vc-only and Vc + Hbx treatments (Fig. 5C, Table S2), becoming a relatively minor constituent of the microbiome (<10%) by 96 h (Table S1).

DESeq2 analysis revealed that Vc-only and Vc + Hbx coral microbiomes shared many of the same differentially abundant taxa (Fig. 5B, Table S2). 19 orders were significantly enriched in both treatments at either 24 or 96 h (Fig. 5B  ii), and eight orders at both 24 and 96 h (Fig. 5B  i). 10 orders were enriched only in the Vc + Hbx treatment compared to the Control treatment, one at 24 h (Rhodospirillales) and nine at 96 h (Fig. 5B  iv). Four orders were significantly enriched in Vc + Hbx corals at 24 h, and both Vc-only and Vc + Hbx corals at 96 h (Fig. 5B  iii).

### *Vibrionales* and *Halobacteriovorax* are rapidly lost from *A. cervicornis* microbiomes

We examined the relative abundance of *Vibrionales*. All ASVs within this order belonged to the *Vibrionaceae* family, however because the V4–V5 regions of the *Vibrionaceae* 16S rRNA gene are highly conserved, we did not distinguish below the family level [89], thus we refer to all *Vibrionaceae* ASVs as *Vibrionales* for consistency. We detected *Vibrionales* ASVs in at least one sample in each treatment group at 0 h, indicating these bacteria were present prior to experimental manipulation (Table S1). At 24 h, *Vibrionales* increased by four orders of magnitude in Vc-only and Vc + Hbx treatments (Fig. 5A and B), and returned to near pre disturbance levels by 96 h (Table S1). *Vibrionales* relative abundance showed unexpected high variability between individual coral fragments within the Vc + Hbx treatment. This may be an artifact of relative abundance data, as *Vibrionales* relative abundance did not correlate with Coral Health Chart assessment.

**Figure 6:**
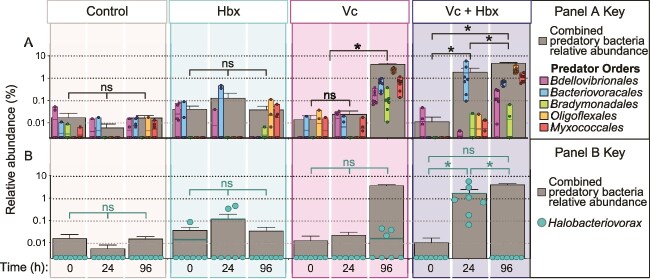
*Halobacteriovorax* does not persist in *A. cervicornis* microbiomes. Relative abundance (%) of the predatory bacteria within microbiome samples from the *Vibrio-Halobacteriovorax* inoculation experiment (Fig. 3). Data are shown as combined predatory bacteria relative abundance (grey bars in A and B) where each bar is the total predator relative abundance averaged across 6–7 coral fragments per treatment per timepoint (*n =* 83). (A) Data from individual predator orders are shown where floating boxes represent relative abundance averaged across 6–7 coral fragments per treatment per timepoint and colored circles indicate values from individual coral fragments. (B) Data from only *Halobacteriovorax* spp. are shown as individual teal circles for each coral fragment. Circles on the x-axis indicate that taxon was below the limit of detection (0.0022%). Asterisks indicate *P <* .0001 using a 2way ANOVA with a Tukey’s multiple comparisons test comparing predator order relative abundance between times within a given treatment (A) or *Halobacteriovorax* relative abundance between times within a given treatment (B). Ns indicates “not significant.” Error bars indicate standard deviation.

We detected five predatory bacterial orders within our samples that made up a small portion of the initial *A. cervicornis* microbiome (averaging 0.021 ± 0.027% across all 0 h samples) (Fig. 6A). Predator relative abundance in Control and Hbx-only samples did not significantly change throughout the experiment, however in Hbx-only samples the composition of predators did shift from *Bdellovibrionales* and *Bacteriovoracales* dominated at 0 h and 24 h to *Oligoflexales* and *Myxococcales* dominated at 96 h (Fig. 6A and S2). In Vc-only samples, predator relative abundance increased by more than 150-fold between 24 and 96 h, from 0.025 ± 0.021% to 3.92 ± 0.76%, respectively. This increase was driven by significant increases in *Oligoflexales* (below the limit of detection [BLD] to 2.41 ± 0.49%), *Bacteriovoracales* (0.0067 ± 0.0074% to 0.59 ± 0.29%), and *Myxococcales* (0.0059 ± 0.0061% to 0.62 ± 0.35) (Fig. 6A and S2).

Within Vc + Hbx corals, predator relative abundance significantly increased from 0 to 24 h, and from 24 to 96 h (Fig. 6A). *Halobacteriovorax* was the only genus to increase between 0 and 24 h (BLD to 1.7 ± 2.14%), however it was completely lost from the microbiome by 96 h (BLD) (Fig. 6B). The increase in predator abundance at 96 h was driven by *Oligoflexales* (0.0057 ± 0.0073% to 1.1 ± 0.45%) and *Myxococcales* (0.0054 ± 0.0084% to 2.37 ± 0.78%) (Fig. 6A and S3). Predator richness followed the same pattern as predator relative abundance, increasing significantly within the Vc-only treatment from 24 (5.9 ± 11.1 ASVs/sample) to 96 h (33.3 ± 5.1 ASVs/sample) and in the Vc + Hbx treatment throughout the experiment (0.4 ± 0.5 ASVs/sample to 7.3 ± 4.3 ASVs/sample to 31.7 ± 11.4 ASVs/sample) (Fig. S3).

## Discussion

This work demonstrates that marine predatory bacteria can halt bacterially induced disease progression in an animal host, suggesting microbial predators have potentially powerful therapeutic applications. Our findings reveal that natural and inoculated predators rapidly respond to disturbed microbiomes, supporting their putative role as top-down ecosystem regulators and natural animal defense systems.

Our observation that *Halobacteriovorax* and overall bacterial predator relative abundance increased dramatically within all *Vibrio*-treated corals, yet not *Halobacteriovorax* only-treated corals, suggests several dynamics are likely at play. First, although the same number of *Halobacteriovorax* cells were added to the Hbx-only and Vc + Hbx treatments, *Halobacteriovorax* was detected in only two out of seven fragments in the predator only treatment at 24 h; all Bdellovibrionota were depleted and replaced by other predator taxa by 96 h. This suggests that *Halobacteriovorax* only established and occupied a niche within these microbiomes when sufficient prey were present, which only occurred after *V. coralliilyticus* RE22 disrupted the microbiome. This crash in the predator population fits a classic predator–prey dynamic, where our experimental increase in predators depleted a specific prey population such that the predator community could no longer be supported. Second, although *Halobacteriovorax* was able to carve out a niche within disrupted samples (Vc + Hbx), it did not persist within this niche and was instead replaced by other predatory taxa within only a few days. *Halobacteriovorax* could have been lost due to a depletion of “ideal” prey (*Vibrionales*), and/or competition by other predators, possibly those better suited to *A. cervicornis* communities. Overall predator abundance was similar between Vc-only and Vc + Hbx corals (~4% of the microbiome), and *Peredibacter* reached similar relatively high abundance to *Halobacterivorax* within Vc-only and Vc + Hbx corals, 0.3% and 0.4% respectively, suggesting a possible carrying capacity for *Bacteriovoracales*, and predatory bacteria in general, within these ecosystems. Because corals within these treatments had very different health statuses at 96 h, this observation suggests that these additional predators appeared to respond to microbiome disturbance without meaningfully altering disease progression. Future studies are needed to determine whether the activity of native predators can prevent disease onset within natural microbiomes. *Peredibacter* has been identified in both healthy [90–92] and dysbiotic [92–94] coral microbiomes, yet little is known about their prey preferences as well as distribution and function in marine environments [95, 96], warranting further exploration of this understudied taxon.

Because of the relatively short experimental duration, the efficacy of predatory bacteria to not only halt but reverse bacterially induced coral disease warrants further investigation. In a previous study, simultaneous inoculation of *Halobacteriovorax* PA1 and *V. coralliilyticus* BAA 450 prevented microbiome shifts in *M. cavernosa* [49], suggesting *Halobacteriovorax* can potentially prevent disease onset if administered prophylactically. Our data suggest inoculated *Halobacteriovorax* cells may temporarily occupy a subset of treated corals, yet do not become long-term residents. This observation is consistent with other probiotic inoculation studies noting probiotic taxa do not typically become long-term symbionts even when providing a benefit to the holobiont [97–99]. Future studies evaluating the impact of subsequent inoculations on disease reversal and long-term retention of *Halobacterivorax* should be performed.

Further investigation should examine the role of predation within coral microbiomes, as well as the details of when and where microbial predation occurs within coral tissue. We did not observe significant community structure shifts in healthy *Halobacteriovorax*-treated corals which was not entirely surprising as these microbiomes were dominated by *“Candidatus”* Aquarickettsia, leaving known Bdellovibrionota prey either low abundance or below the limit of detection in these samples. *A. cervicornis* ML-50 was previously characterized as a disease-susceptible genotype [62] dominated by *“*Ca.*”* Aquarickettsia [63], an obligate *Rickettsiales* parasite linked with reduced *A. cervicornis* fitness. Although this taxon could be susceptible to predation, as it is gram-negative, this is unlikely given its intracellular lifestyle; Bdellovibrionota tend to target extracellular bacteria [100], eliminating the complications of invading host cells for prey access.

Our observations raise many questions regarding the mechanistic underpinning of *V. coralliilyticus-*induced disease in *A. cervicornis*. *V. coralliilyticus* RE22 affected multiple components of the coral holobiont in our experiment including the bacterial community, endosymbiotic dinoflagellate, and host cells themselves manifested as microbiome restructuring, decreased pigmentation, and tissue sloughing, respectively. These changes could be a result of direct antagonism by *V. coralliilyticus* RE22 against one of these organisms (i.e. only host cells) which caused cascading effects to the rest of the holobiont, or alternatively against multiple or all of these organisms independently [30, 31, 33, 88, 101, 102]. For example, a recent study revealed that multiple *V. coralliilyticus* strains can employ both antieukaryotic and antibiotic activity via two distinct type VI secretion systems (T6SSs) [103]. Using culture-based assays, an invertebrate model (*Artemia salina)*, and proteomics, researchers elegantly determined that these molecular weapons were highly conserved among *V. coralliilyticus* strains and their expression is temperature-dependent. Future work should explore whether *V. coralliilyticus* RE22 T6SSs or other molecular mechanisms contribute to virulence in *A. cervicornis* and if so, what component(s) of the coral holobiont they target.

Our data are consistent with the hypothesis that coral diseases progress in successional stages [27, 93], whereby primary pathogen(s), in this case *V. coralliilyticus* RE22, disrupt and destabilize the microbiome allowing uncontrolled growth of native microbes, both opportunists and beneficial symbionts, as well as foreign invaders. We observed a significant decrease in *“*Ca.*”* Aquarickettsia and increase in copiotrophic opportunists, specifically Alteromonadales and Flavobacterales, in *Vibrio*-treated corals, similar to previous temperature-induced bleaching studies on this same coral genotype [63, 64]. Other taxa that increased within our *Vibrio*-treated corals have been implicated in SCTLD [104, 105] and type II white band disease [77]. However, detailed mechanistic studies are required to fully support the hypothesis that negative host outcomes are due to both the activity of *V. coralliilyticus* and taxa that take advantage of disrupted microbiomes. Further, whether more ecologically relevant *V. coralliilyticus* strains induce disease phenotypes in successional stages in Caribbean *Acropora* spp., requires additional exploration.

In conclusion, our findings highlight the potential of *Halobacteriovorax* to halt disease progression in stony corals. Further research is needed to explore the long-term efficacy, scalability, and potential use of this taxon to treat and prevent natural diseases plaguing restoration and wild coral colonies, particularly polymicrobial infections.

## Supplementary Material

HHD_supplemental_documents_sub2_wraf270

## Data Availability

16S rRNA sequence data are available on NCBI under Bioproject PRJNA1291262; housekeeping gene data for heterotrophic strains are available on NCBI under submission ID 3014808. Code for this work can be found at https://github.com/spearel/Acer-Vibrio-Hbx-Mote2023.
