# Supplementary material for: Halobacteriovorax halts disease progression in endangered Caribbean corals
Source: ISME J. 2025 Dec 10;19(1):wraf270. doi: 10.1093/ismejo/wraf270 (PMC12747082; doi:10.1093/ismejo/wraf270)
Supplement: HHD_supplemental_documents_sub2_wraf270 [file hhd_supplemental_documents_sub2_wraf270.docx]

**Supplemental Tables:**

**Table S1.** Relative abundance of top ten orders within Vc-only and Vc-Hbx *A. cervicornis* ML-50 microbiomes. Data are reported as Average abundance +/- standard deviation with 6-7 biological replicates per time and treatment. ’Cyano’ indicates Cyanobacteria.

**
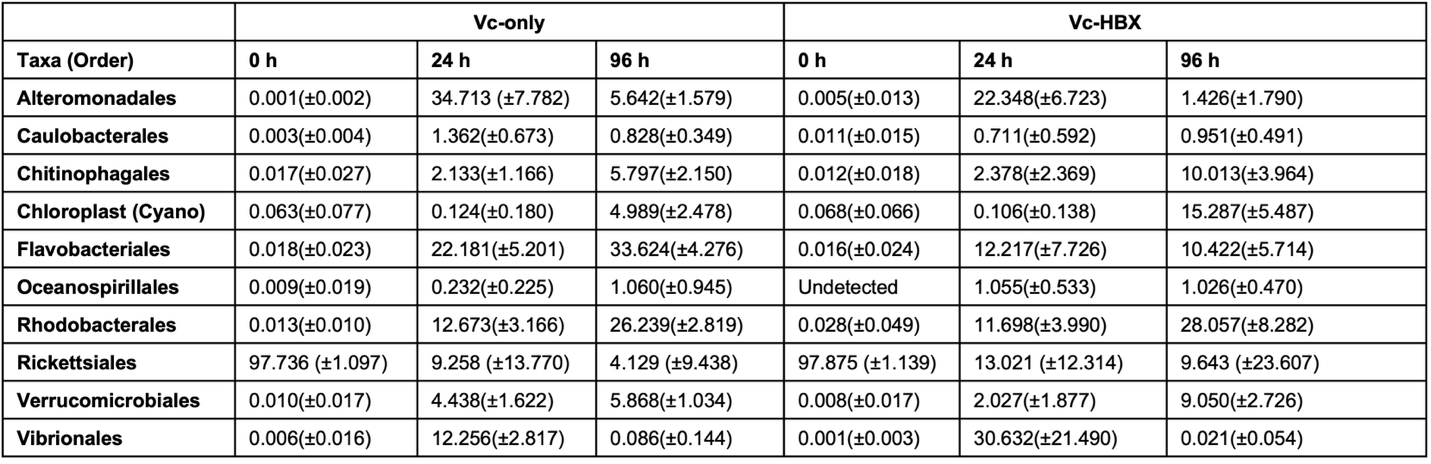
**

**Table S2**. Order-level DESeq2 analysis of differentially abundant (DA) taxa. Data are subsampled by time and assessed by comparing each treatment group to the no inoculation control. Column headings are as follows: DA Order, baseMean, log2FoldChange, IfcSE, stat, pvalue, padj, and comparison groups. **table not attached for size.*

**Supplemental Figures:**

**
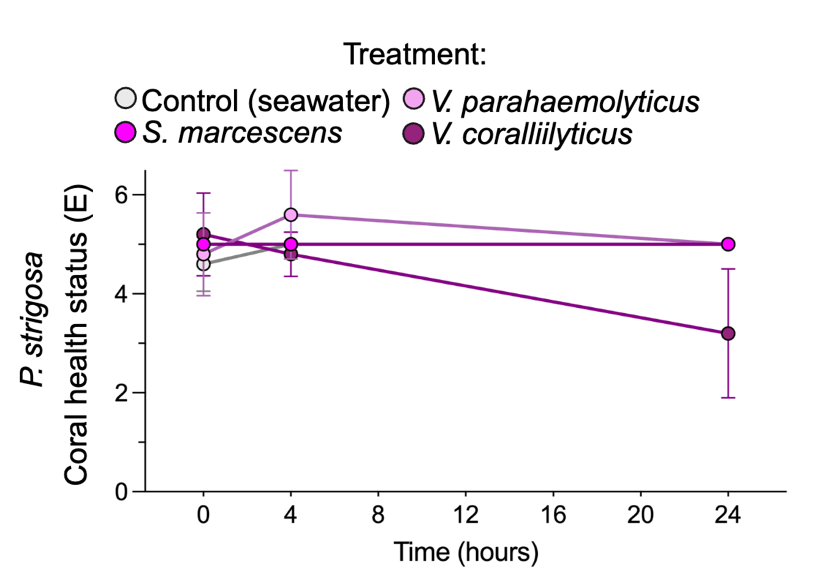
**

**Figure S1. *V. coralliilyticus* does not significantly affect *P. strigosa* coral health status.** Corals were exposed to sterile seawater (control; gray), or one of three bacterial cultures: *S. marcescens* (medium), *V. parahaemolyticus* (light), or *V. coralliilyticus* (dark). Data are shown as coral health status (B: *P. strigosa; D*: *A. cervicornis*). Two-way ANOVA with Dunnett’s multiple comparison test did not indicate any statistically significant difference between any bacterial inoculum versus the control. Five coral frags were used in each treatment (n=20); error bars indicate SEM.

**
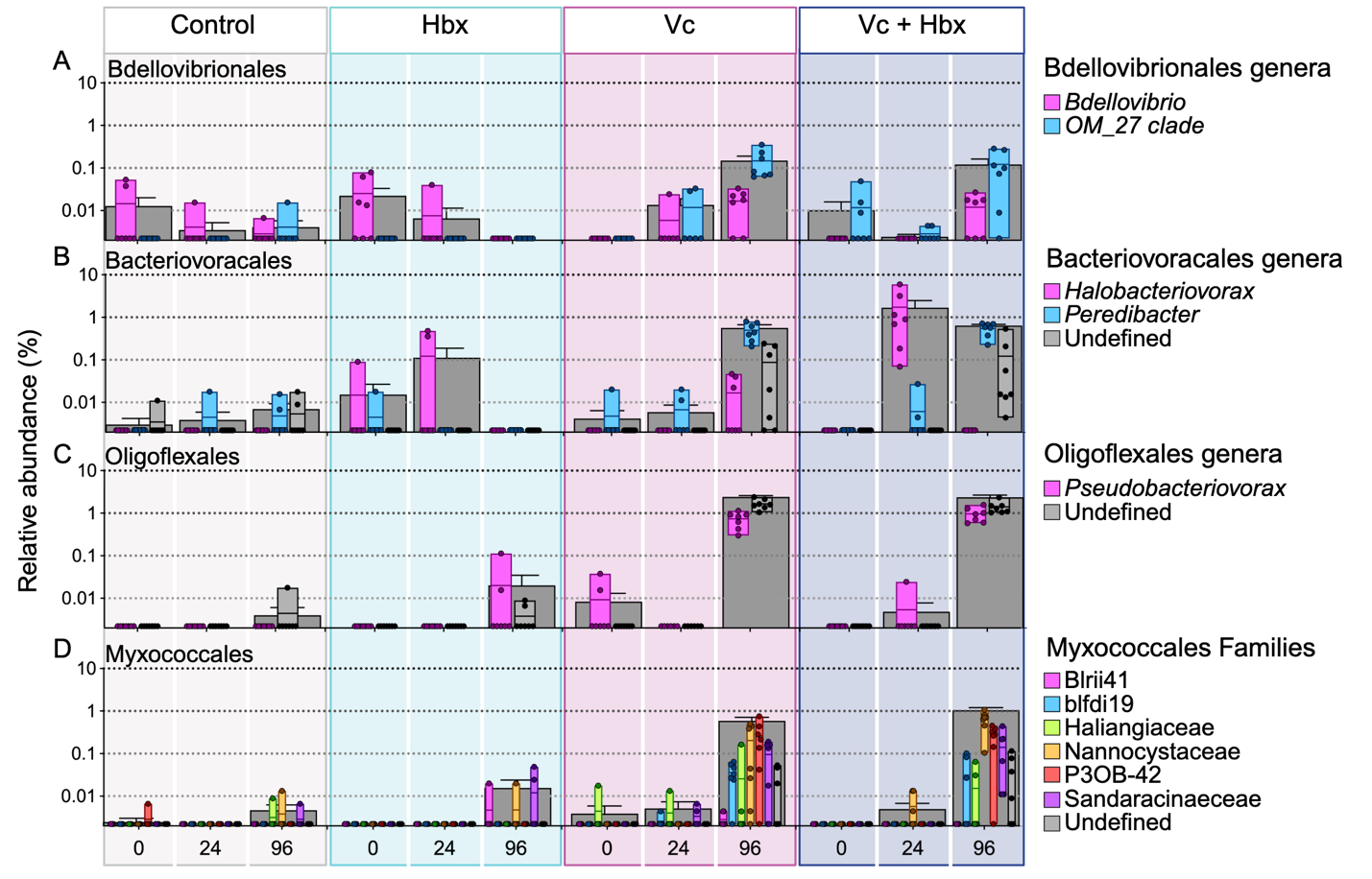
**

**Figure S2. *V. coralliilyticus* and *Halobacteriovorax* shift microbial predator community structure.** Relative abundance (%) of microbiome samples from the *Vibrio-Halobacteriovorax* inoculation experiment. Data are shown as the sum of a given order (gray box) with the constituent bacterial family or genera shown in colored boxes; circle symbols indicate values for each coral fragment within a treatment (n=83). The x-axis begins at the limit of detection (0.0022%). Error bars indicate standard deviation.


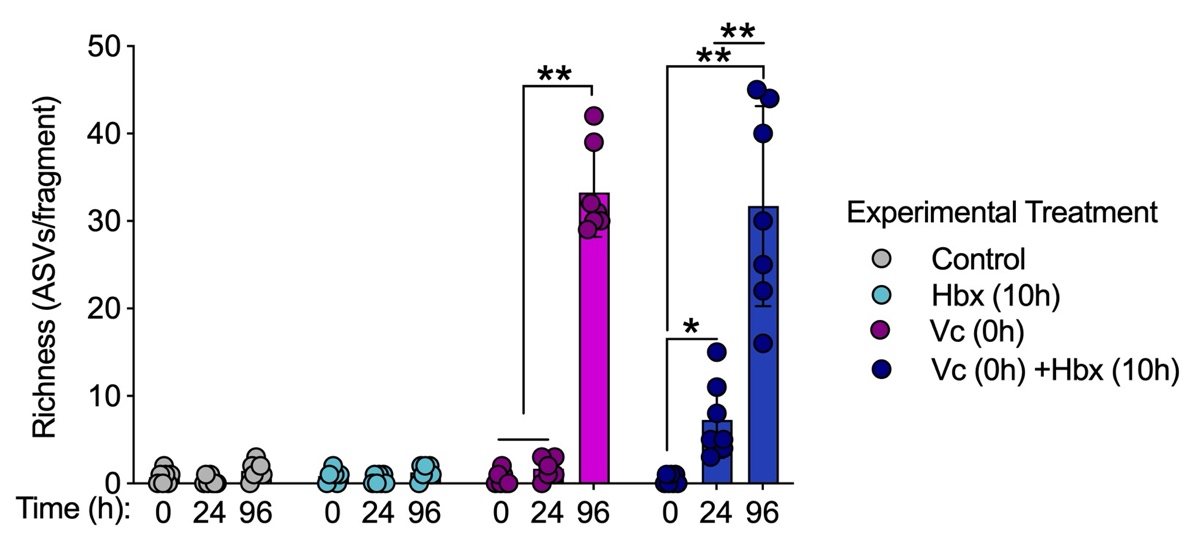


**Figure S3. Predator bacterial richness.** Alpha diversity of the predatory bacterial community within *A. cervicornis* microbiome samples from the *Vibrio-Halobacteriovorax* inoculation experiment. Data are shown as Richness (number of ASVs per coral fragment per treatment per time point) where each circle represents an individual coral fragment (n=83). Circle color and bar color indicate the experimental treatment. Asterisks indicate P*<0.01* (*) or P*<0.0008* (**) for a mixed-effects analysis and Tukey’s multiple comparisons comparing predator richness between times within a given treatment; error bars indicate standard deviation.
